# Fusion Graph Representation of EEG for Emotion Recognition

**DOI:** 10.3390/s23031404

**Published:** 2023-01-26

**Authors:** Menghang Li, Min Qiu, Wanzeng Kong, Li Zhu, Yu Ding

**Affiliations:** 1 College of Computer Science and Technology, Hangzhou Dianzi University, Hangzhou 310018, China; 2 Key Laboratory of Brain Machine Collaborative Intelligence of Zhejiang Province, Hangzhou 310018, China; 3 Netease Fuxi AI Lab, Hangzhou 310018, China

**Keywords:** emotion recognition, EEG, graph convolutional network, feature fusion

## Abstract

Various relations existing in Electroencephalogram (EEG) data are significant for EEG feature representation. Thus, studies on the graph-based method focus on extracting relevancy between EEG channels. The shortcoming of existing graph studies is that they only consider a single relationship of EEG electrodes, which results an incomprehensive representation of EEG data and relatively low accuracy of emotion recognition. In this paper, we propose a fusion graph convolutional network (FGCN) to extract various relations existing in EEG data and fuse these extracted relations to represent EEG data more comprehensively for emotion recognition. First, the FGCN mines brain connection features on topology, causality, and function. Then, we propose a local fusion strategy to fuse these three graphs to fully utilize the valuable channels with strong topological, causal, and functional relations. Finally, the graph convolutional neural network is adopted to represent EEG data for emotion recognition better. Experiments on SEED and SEED-IV demonstrate that fusing different relation graphs are effective for improving the ability in emotion recognition. Furthermore, the emotion recognition accuracy of 3-class and 4-class is higher than that of other state-of-the-art methods.

## 1. Introduction

Emotion recognition is a human–computer interface task based on multiple modalities, e.g., facial expressions, audio tunes, and psychological signals. Among them, psychological signals can be difficult to disguise or hide. Moreover, EEG-based emotion recognition draws more attention due to its portability and low equipment cost. CNN-based methods are generally used to extract emotional features from EEG for classification tasks. Recently, Li et al. [1] captured temporal and spectral descriptors through squeeze and excitation operations for tasks based on EEG. Existing studies based on CNN only considered the signal of euclidean-distributed electrodes and failed to explore the complex brain connectivity between different electrode sites. However, studies from neuroscience have shown that the spacial relationship of non-euclidean distributed electrodes can also provide important clues for studying brain function. Researchers developed various graph neural network (GNN) models to overcome this limitation by projecting EEG electrodes onto the graph nodes, updating graph edge weights, and applying the graph to EEG-based tasks. Studies on topology connection [2,3,4,5,6], functional connection [7,8,9,10,11,12,13], and effective connection [14,15,16,17,18,19,20,21] have proved their superiority in extracting helpful information on channel relationships for EEG emotion recognition. Topology connection is to measure the adjacency of channels in physical distance. Papers [2,3] used the topological relationship between EEG channels to construct an adjacency matrix. Applying that matrix to a graph convolution model can improve its performance more than randomly initializing the graph. Functional connectivity measures the statistical dependence of signals in time or spectral space. The use of functional connectivity between EEG channels in a graph convolution model has also provided practical information and achieved good results in emotion classification [22]. Effective connectivity measures the causal relationships of signals in time or spectral space. Functional connection measures the statistical dependency of signals in time or spectral space. Using the functional connection between EEG channels in a graph convolution model has also provided useful information and achieved good results in emotion classification [22]. Effective connection measures the causal relations of signals in time or spectral space. Considering the effective connection can improve the accuracy of EEG-based emotion recognition because the efficacious connection between EEG channels is consistent with the characteristics of EEG laterality. The causal discovery of time series data helps to interpret data and is crucial for the rapidly evolving field of explainable artificial intelligence [23]. However, existing graph-based methods examine one connection feature simultaneously, not considering all connection features yet. Thus, they need comprehensive information between channels. Based on the above research, the adjacency matrices constructed by the existing graph convolution models have the problem that their utilized connection feature is too simple to capture comprehensive information. To further optimize the graph-based model and extract the diversified information between channels, we build a fusion graph convolution model, which considers the topological connection, effective connection, and functional connection of EEG signals simultaneously. We test the model on 3-class EEG-based emotion recognition to show its superior performance.

The main contributions of the paper are as follows:

- We propose the fusion connection of EEG signals for the first time, combining topological, functional, and effective connections, which proves its effectiveness in feature extraction.
- We propose a unified and generalizable architecture for fusion graph convolution, which proves its robustness and effectiveness in EEG emotion recognition.
- Extensive experiments are conducted on two benchmark datasets for 3-class and 4-class EEG-based emotion recognition. The experimental results show that our FGCN consistently outperforms all state-of-the-art models.

## 2. Related Work

EEG data consists of different connection features, including topological, functional, and effective connections. Studies on the graph concentrate on constructing the connection of channels because of its property in processing data in non-Euclidean space. Universal brain connections are shown in this section.

### 2.1. Topological Connection

Papers [2,3,4,5,6] utilize the topology structure to construct the graph. Jang et al. [2] used the physical distance between EEG electrodes to obtain an adjacency matrix containing intra-band and inter-band connections, successfully represented EEG data as a graphed signal, and applied it to video recognition based on EEG. Zhong et al. [3] considered the biological topological structure among different brain regions to capture local and global relationships between EEG channels, constructing an adjacency matrix of graph convolution. Introducing graph theory to brain networks, Chen et al. [4] used a minimum spanning tree to generate a topology graph according to link strength. Paper [5] considered brain topology metrics based on graph theory, which determined local and global efficiency. Duan et al. [6] considered the number of steps required to get from one node to another in a brain network, and defined the average of the shortest paths between any two nodes in the network as the topological connection.

### 2.2. Functional Connection

The following studies utilize functional correlation to construct the graph. Using functional correlation to initialize the adjacency matrix, Song et al. [9] dynamically updated a matrix during the graph convolution operation to improve emotion recognition accuracy. Wang et al. [10] used Phase Lock Value (PLV) to model multi-channel EEG signal features as graphic signals to extract inter-band information implicit in EEG signals. GCNs-Net [11] introduced the absolute Pearson matrix of the overall signal to distinguish four types of mental imagery intentions by establishing the Laplacian graph of an EEG electrode. The results proved that the method could converge personalized and group predictions. EEG-GNN [12] considers functional neural connectivity to construct a sparse graph, which is critical for reducing computational costs and designing portable EEG headsets, and considers five types of functional connectivities to construct a brain network. The network consists of nodes and edges, where each EEG electrode is defined as a node, and the edges represent the connectivity strength between different EEG electrodes.

### 2.3. Effective Connection

Some studies [14,15,16] have proved that effective connection may exist in brain activities. Sohrabpour et al. [14] proved that Granger causality analysis is potent for studying effective connection. Herrmann et al. [15] demonstrated that the relationship between oscillations of brain activity and cognitive processes is causal. Hesse et al. [16] proved that the actual mutual influences between any two nodes are different. Learning the directional connections between brain regions can effectively study brain conditions and improve the accuracy of EEG-based emotion recognition. Paper [17,18,19,21] utilized the causal correlation to construct graphs. Uchida et al. [17] used Granger causality and graph theory to analyze the EEG data of epileptic patients with VNS. Hejazi et al. [18] investigated how effective connectivity changes the effect on unexpected seizure prediction. Hosseini et al. [19] identified the effective connectivity between active cortical regions during mental fatigue with visual stimulation and presented a dynamic causal model. Kong et al. [21] applied Granger causality analysis to extract the effective connectivity between pairwise channels and improve the accuracy of EEG-based emotion recognition in 2022.

Previous work did not consider them simultaneously, resulting in information loss in extracting brain connection features. This paper proposes fusion graph convolutional networks to fix that problem by fusing diverse brain connections. After the fusion of those representations, we can obtain the universal representation of EEG. Our experiments have verified the superiority of the fusion graph on 3-class and 4-class EEG-based emotion recognition.

## 3. Method

**Figure 1:**
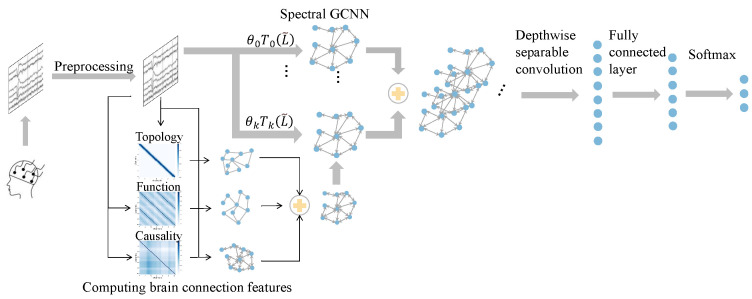
The flow chart of FGCN. $\theta_{k}T_{k}\left(\tilde{L}\right)$ denotes the Chebyshev polynomials and θ is updated during the process of graph convolutional neural network.

We first propose the fusion graph to represent the pair relationship between EEG channels using EEG data as the input. Then, we present the fusion-graph-based convolutional neural network to fully utilize the extracted information and classify different emotional states. This section mainly introduces the way of representing the fused brain connection features proposed in our method. On this basis, we propose our unified architecture for EEG-based emotion recognition, a fusion-graph-based convolutional neural network (FGCN). Finally, we show the way to optimize our method. The overall flow of the proposed method is shown in Figure 1.

### 3.1. Graph Construction

Herein, we introduce how to construct different connection graphs and fuse them.

#### 3.1.1. Topological Graph Construction

**Figure 2:**
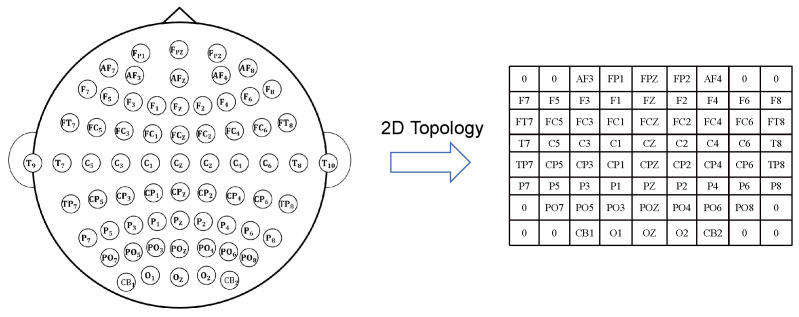
Two-dimensional coordinates of the 62 EEG channels. We project the 62 electrodes of the EEG into 2D. The positions of channels are shown on the left and resized into an 8 × 9 matrix.

Salvador et al. [24] observed that the strength of the connection between brain regions decays as an inverse square function of physical distance. Based on that, constructing the topology graph is one way to capture brain connectivity features. The adjacency matrix $A_{T}\in R^{N\times N}$ of the topology graph represents the topological structure of EEG channels, where *N* is the number of EEG channels. Each element $A_{ij}^{T}$ indicates the weight of the connection between channels *i* and *j*. According to the position of EEG channels, we visualize the position matrix as shown in Figure 2. Moreover, we initialize the topological relations using the Radial Basis Function (RBF) to obtain its mathematical representation, as shown in Equation (1).

(1) $$A_{ij}^{T}=exp\left(-\frac{{\left[dist\left(i,j\right)\right]}^{2}}{2\theta^{2}}\right)$$

where dist(·) represents the Euclidean distance between channels *i* and *j*, and the constant θ controls the radial range of action.

#### 3.1.2. Functional Graph Construction

The functional graph considers the correlation between channels describing linear coherence between two variables (time series). We consider the functional graph for EEG data since EEG signals are time continuous, and each channel is relatively independent. The Pearson coefficient is based on the covariances and then divided by their standard deviations, and fixes some problems in covariance such as high time consumption and computation cost. Adopting the Pearson correlation coefficient to obtain the correlation information between channels is most suitable for our downstream task. The adjacency matrix $A_{F}\in R^{N\times N}$ of the functional graph represents the function connection of EEG channels, where *N* is the number of EEG channels. Each element $A_{ij}^{F}$ indicates the weight of the connection between channels *i* and *j*. Equation (2) defines the matrix containing the correlation information as

(2) $$A_{ij}^{F}=corr\left(i,j\right)=\frac{cov(i,j)}{\sigma_{i}\sigma_{j}}$$

where cov(i,j) represents the covariance of channels *i* and *j*; $\sigma_{i}$ and $\sigma_{j}$ represent the product of their standard deviations, respectively.

#### 3.1.3. Causal Graph Construction

Asymmetry is an essential descriptive feature of effect, reflecting the connectivity features that one variable will change when other variables change. Studies have shown a significant relationship between asymmetric brain activity patterns and emotional states.

Granger causality [25] for inferring time series causality expresses the intensity of a causal relation between time series. In order to measure it, researchers proposed the GC test [26] as a measurement method of Granger causality in 1980. It is generally accepted and widely used to illustrate information interactions between time series via the GC test. Thus, we use the GC test to obtain causal relationships between EEG channels. The prediction error is calculated between channels to construct the causal graph, which reflects the information flow between brain regions.

We construct a causal graph to obtain insight into the underlying mechanisms of brain activity. The adjacency matrix $A_{C}\in R^{N\times N}$ of the functional graph represents the function connection of EEG channels, where *N* is the number of EEG channels. Each element $A_{ij}^{C}$ indicates the weight of the connection between channels *i* and *j*. Equation (3) describes the matrix containing the correlation information,

(3) $$A_{C}=\left\{\begin{array}{cc} A_{ij}^{C}=GC_{i\leftarrow j}=\ln\frac{\sigma_{i}^{2}}{\sigma_{ji}^{2}} & \\ A_{ji}^{C}=GC_{j\leftarrow i}=\ln\frac{\sigma_{i}^{2}}{\sigma_{ji}^{2}} & \\ A_{ii}^{C}=1 & \end{array}\right.$$

where $\sigma_{i}$, $\sigma_{ij}$, $\sigma_{ji}$, and $\sigma_{j}$ mean the prediction error variance that are defined in the GC test. $A_{ij}^{C}$ denotes the causal factor calculated using Granger causality, representing the causal relations from channel *i* to channel *j*.

### 3.2. Graph Fusion Strategy

Graph fusion strategy aims to help the strong connections presented in one or more graphs to enhance and the weak connections to disappear simultaneously, thus reducing the noise in the fusion graph. The local fusion strategy is proposed based on the assumption that local connections with high similarities are more reliable than non-local ones.

After the graph construction step, we have M(M=3) graphs with the same nodes but different edges. They represent three types of connection matrices $A_{m}\in R^{N\times N}\;\left(1\leq m\leq M\right)$. To fuse these connection matrices is to obtain a universal representation of EEG data to recognize emotional states. First, we apply a normalization step to all connection matrices. The usual normalization may not be numerically stable since it ignores self-similarities in the diagonal entries of $A_{m}$. Thus, we perform the normalization over the row $A_{i}^{m}\left(1\leq p\leq N\right)$ of the connection matrix $A_{m}$. Equation (4) describes the process of the normalization operation as follows:

(4) $$H_{i}^{m}=\left\{\begin{array}{cc} H_{ij}^{m}=\frac{1}{2}\times \frac{A_{ij}^{m}-{\left(A_{i}^{m}\right)}_{min}}{{\left(A_{i}^{m}\right)}_{max}-{\left(A_{i}^{m}\right)}_{min}}, & j\neq i\\ H_{ij}^{m}=1/2, & j=i \end{array}\right.$$

To avoid overfitting the proposed model, we also use a hard threshold to sparse each type of connection matrix $H_{m}$. Then, we consider the position factor of causal or functional connections to obtain connections with more prosperous and more reliable information for representing EEG data. Thus, we explore a local fusion strategy in fusing graphs, implemented by point-by-point addition. As shown in the section on ablation study, though we tried the other four fusion strategies, their results are worse than point-by-point addition. Equation (5) describes how to fuse different spatial relationships of EEG data.

(5) $$H_{ij}^{fuse}=H_{ij}^{T}⊕H_{ij}^{C}⊕H_{ij}^{F}$$

where $H_{ij}^{T}$, $H_{ij}^{C}$, and $H_{ij}^{F}$ represent values on position (i,j) of the causal graph, functional graph, and topological graph, respectively.

### 3.3. Fusion Graph Convolutional Neural Network

Graph convolutional network has more advantages when processing signals than CNN and considers the relationship between EEG channels to extract the spatial features of nodes [27]. Herein, the topological, functional, and causal relationships are considered and fused as the fused graph. Then, the fused graph and original EEG signals are the input of the graph convolutional network. Equation (6) denotes the proposed spatial GCN in the paper [27],

(6) $$H^{l+1}=\sigma \left(D^{-1/2}H_{fuse}D^{-1/2}O^{l}Wl\right)$$

where *D* denotes the diagonal degree matrix of $H_{fuse}$. The normalized adjacency matrix $D^{-1/2}H_{fuse}D^{-1/2}$ prevents H from growing overly large. *l* denotes the number of layers. *O* and *W* are the outputs and parameters of the *l*th graph convolution layer. Due to DE and other features of EEG data being spectral features, the spectral GCN [28] involving graph Fourier transform is more fittable for our task. Thus, the spectral GCN is adopted as the backbone of FGCN. The version we use was proposed by Defferrard et al. [27] and uses Chebyshev polynomials to approximate the filtering operation. Equation (7) is the expression of the spectral GCN.

(7) $$X*G_{fuse}=U\hat{G}U^{T}X\approx \sum_{i=0}^{k}\theta_{i}T_{i}R_{fuse}^{\prime }X$$

where $T_{i}\left(\cdot \right)$ denotes the Chebyshev polynomials, $\theta_{i}$ denotes learnable parameters, and $R_{fuse}^{\prime }$ is the scaled normalized Laplacian with its eigenvalues lying within [−1,1]. Equation (8) shows the way to compute $R_{fuse}$ and Equation (9) shows the way to compute $R_{fuse}^{\prime }$, as follows:

(8) $$R_{fuse}=I_{N}-D^{-1/2}H_{fuse}D^{-1/2}$$

(9) $$R_{fuse}^{\prime }=\frac{R_{fuse}}{\lambda_{max}-I_{N}}$$

where *N* is the number of nodes in the fusion graph and λ is the maximum eigenvalue of $R_{fuse}$. Depthwise separable convolution can significantly reduce model calculation amounts and operation times compared with ordinary convolution. To make the model more efficient, we combine depthwise separable convolution and graph convolution to extract discriminative EEG signal features further. Furthermore, label prediction is implemented through a fully connected layer with softmax activation. We use cross entropy as the loss function in this paper. Equation (10) shows the loss function as follows:

(10) $$L=-\sum_{s=1}^{N}\sum_{k=1}My_{sk}\log p(\hat{y_{sk}})$$

where $y_{sk}$ is a binary indicator meaning the label of sample *s* is *k* and $p(\hat{y_{sk}})$ represents the probability that the label prediction of sample *s* is correct.

## 4. Result and Analysis

### 4.1. Datasets

We conduct experiments on the SEED dataset and SEED-IV dataset. The SEED dataset [29] comprises EEG data of 15 subjects recorded in 62 channels. The data were collected while participants watched stimuli movies with three emotions: negative, neutral, and positive. Each movie lasts around 4 min. Three data sessions were collected, each containing 15 trials/movies for each subject. To make a fair comparison with existing studies, we directly use the pre-computed differential entropy (DE) features, differential asymmetry (DASM) features, and differential caudally (DCAU) features in SEED. In SEED, the upper three features are pre-computed over five frequency bands (δ,θ,α,β, and γ) for each second of EEG signals in each channel. In the experiment, we use the first nine trials as the training set and the remaining six as the test set. We average the results of 15 subjects to obtain the eventual accuracy and variance.

The SEED-IV dataset [30] comprises EEG data of 15 subjects recorded in 62 channels. The data were collected when participants watched stimuli movies with four emotions: sad, fearful, neutral, and positive. Three data sessions were collected, comprising 72 trials/movies for each subject. To make a fair comparison with existing studies, we directly use the pre-computed differential entropy (DE) features in SEED-IV. In SEED-IV, DE features are pre-computed over five frequency bands (delta, theta, alpha, beta, and gamma) for each second of EEG signals in each channel. In our experiment, we use the first 15 trials as the training set and the remaining nine as the test set. We average the results of 15 subjects to obtain the final result. In this experiment, we quantitatively evaluate the performance of predicting EEG emotion states using the average accuracy and variance of emotion recognition, which reveals the proportion of correct predictions and robustness.

### 4.2. Comparison with Other State-of-Art Methods

For pre-computed differential entropy (DE) features, differential asymmetry (DASM) features, and differential caudality (DCAU) features, experiments were carried out in different frequency bands (δ,θ,α,β,γ, and full band). The proposed model is compared with SVM [31], GCN [27], DGCN [9], R2G-STNN [32], and BiHDM [33] on the SEED dataset with DE features in this paper. SVM is a classic machine learning method, while the others are state-of-the-art. In the method using SVM, EEG features are fed directly into the SVM to predict emotion states. In the graph-based methods, brain features are pre-computed before input into the network. The performance of these graph-based methods is improved, proving that graph convolution is efficient for EEG emotion recognition. However, they only exploit functional information regarding the relationship between EEG channels. Inspired by these methods, we proposed our method of fusing multiple brain features to obtain a unified and generalized feature for emotion recognition.

The specific results are shown in Table 1. GCN is the baseline of the proposed FGCN, with the recognition accuracy achieving 87.40%. DGCN updated GCN by dynamically updating the constructed graph, improving the recognition accuracy to 90.40%. R2G-STNN expanded the spatial relationship from local to global. BiHDM extracted the spatial discrepancy between hemispheres and obtained a recognition accuracy of 93.12%. FGCN fused different graphs containing brain connection features to obtain a unified representation of EEG data and achieve a relatively higher accuracy of 94.1%.

This paper compares the proposed model with SVM [31] and DGCN [9] on the SEED-IV dataset with DE features. Furthermore, the specific results are shown in the right part of Table 1. DGCN improves the 4-class recognition accuracy from 56.61% to 69.88%. BiHDM improves the 4-class recognition accuracy to 74.35%. FGCN obtains a unified representation of EEG data and achieves a relatively higher accuracy of 77.14%. The paper also compares the proposed model with SVM [31], GCN [27], and DGCN [9] on the SEED dataset with DASM and DACU features. Furthermore, the specific results are shown in Table 2 and Table 3. We achieved a higher recognition accuracy under different frequency bands and the total one.

By comparing different features on the SEED dataset, we find that the DE features still have the highest accuracy in emotion recognition, indicating that DE features are most suitable for emotion-related signal processing. In summary, the accuracy of single band and full band has been improved to a certain extent for DE features, which is more evident in single band. For the DASM and DCAU features, the accuracy improves on most bands. Similarly, all three characteristics are high-frequency bands, full bands are more effective than low-frequency bands, and high-frequency band signals contain more emotion-related information than other frequency band signals. Moreover, the standard deviation of the model on the SEED dataset is also reduced to a certain extent, indicating that the individual differences in the fusion graph convolution are relatively small and it is a relatively stable model.

It can be seen from Table 1, Table 2 and Table 3 that the fusion graph convolutional network achieved better performance compared with other graph methods on the SEED and SEED-IV datasets and has broader applicability. Moreover, experiments on different frequency bands have shown that the β band and γ band contain more emotion-related features than other bands. This finding is consistent with findings in former research [9,27,31]. So, the recognition accuracy of these two bands is higher than other frequency bands.

### 4.3. Ablation Study

#### 4.3.1. The Effectiveness of Fusion Graph Representation

**Figure 3:**
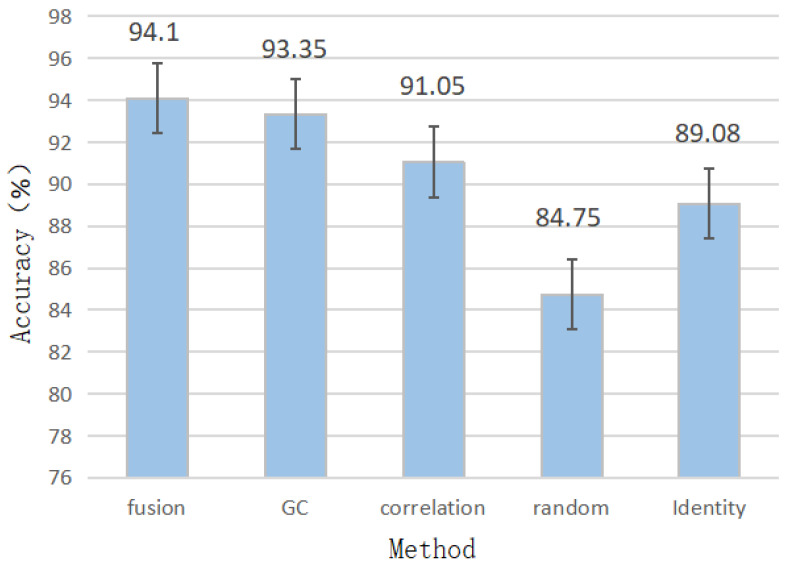
Comparison of different graph construction strategies. The abscissa represents the adjacency matrix used by the model, and the ordinate is the EEG emotion recognition accuracy of the model using the adjacency matrix on the DE features of the SEED dataset.

To verify that the fusion adjacency matrix can provide more helpful information, we conduct ablation experiments on the DE features of the SEED dataset. The experiments compare different performances of the random, identity, correlation, causality, and fusion matrix proposed in this paper. The random matrix is obtained by obeying a uniform distribution in the interval [0,1), the identity matrix is the N×N square matrix with ones on the main diagonal and zeros elsewhere, the correlation matrix is obtained by functional connection, and the causality matrix is obtained via GC test. The results are shown in Figure 3. The abscissa represents different adjacency matrices obtained in upper ways, and the ordinate represents the EEG-based emotion recognition accuracy. Compared with the single use of causality matrix or correlation matrix, the fusion adjacency matrix model is the most accurate for emotion recognition, achieving 94.1%. The accuracy is 0.75% higher than the causality matrix, 3.05% higher than the correlation matrix, 5.02% higher than the identity matrix, and 9.35% higher than the random matrix. Different matrices contain different relationships between EEG channels. Among them, the identity matrix contains self-similarity information of EEG channels. The correlation matrix reveals the functional connection of EEG channels. Moreover, the causality matrix reveals asymmetric information flows between EEG channels. The information extracted by the fusion graph convolution model is more diverse, which should be why the graph convolution accuracy rate using the fused adjacency matrix is higher than that of other information simplification adjacency matrices. With the spatial information of EEG data being more prosperous, the representation of EEG data becomes more accurate. This result also proves that when the information in the adjacency matrix is diversified, the accuracy of the graph convolution model in identifying emotions will be higher.

**Figure 4:**
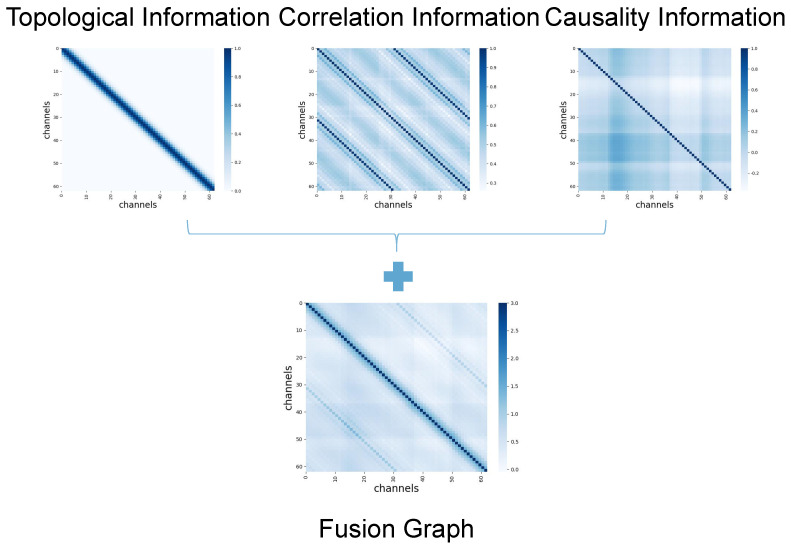
Visualization of fusion graph and other graphs. The fusion graph fuses the topological, functional, and causal graphs. The fusion strategy is point-by-point addition.

In order to more intuitively observe the difference between the fusion adjacency matrix and other matrices, and observe the construction process of the model matrix, this paper visualizes the construction process of the fusion adjacency matrix, as shown in Figure 4. The abscissa and the ordinate represent channels, and the greater the interaction between channels, the darker the color. The adjacency matrix required for the final fusion graph convolution is obtained by adding the topological matrix, correlation matrix, and causality matrix.

It can be seen intuitively that the matrix contains more abundant information than others. When different methods are used, the connections between the channels are different, which also shows that obtaining as much helpful information as possible is necessary to obtain more specific dependencies between the channels.

#### 4.3.2. The Influence of Different Fusion Strategy

It can be seen from Table 4 that the fusion strategy using point-by-point addition can more accurately fuse different information. Furthermore, we have tried other fusion strategies, including the cross-diffusion process, the point-by-point product, the Kronecker product [34], and the Kronecker addition. The model using the point-by-point addition fusion method achieves an accuracy of 94.1% in the entire band of the SEED dataset DE features. The accuracy is 2.32% higher than the model using point-by-point multiplication and fusion, 3.54% higher than the model using Kronecker product, 2.69% higher than the model using Kronecker addition, and 3.74% higher than the model using cross-diffusion process. We can infer that adding matrix elements is better than the multiplication realized. Further, the information transfer among adjacent positions may result in confused recognition. The results prove that when adjacency matrix information is fused, the effect of point-by-point addition is more suitable than other fusion strategies.

## 5. Conclusions

Aiming to unify the adjacency matrix information in the existing graph convolution model, we propose fusion graph convolution, which fuses topological, causal, and functional information. The model first calculates three relationships between EEG channels—topological relationship, functional relationship, and causal relationship—and then uses a local graph fusion strategy based on an addition operator to perform a fusion operation on three graphs containing different brain connection features. Experiments conducted on the SEED dataset show that the proposed fusion graph convolution neural network (FGCN) improves emotion recognition accuracy compared with other graph models. The results illustrate that the fusion graph contains rich spatial information of EEG data and proves the effectivity of FGCN. Experiments on different frequency bands prove that the β and γ band are more effective for EEG-based emotion recognition. The results of the ablation experiments also prove that the more affluent the brain connection information contained in the graph, the more supervising the FGCN is for EEG-based emotion recognition. Meanwhile, adopting point-by-point addition as the local fusion strategy performed better than other fusion mechanisms on EEG-based emotion recognition.

## Figures and Tables

**Table 1:** The accuracy and standard deviation on SEED and SEED-IV with DE features. The best results are marked in bold.

Dataset	SEED						SEED-IV
Classifier	*δ* (%)	*θ* (%)	*α* (%)	*β* (%)	*γ* (%)	Total (%)	Total (%)
SVM	60.50/14.14	60.95/10.20	66.64/14.41	80.76/11.56	79.56/11.38	83.99/09.72	56.61/20.05
GCN	72.75/10.85	74.40/08.23	66.64/14.41	83.24/09.93	83.36/09.43	87.40/09.20	–
DGCN	74.25/11.42	71.52/05.99	73.46/12.17	83.65/10.17	85.73/10.64	90.40/08.49	69.88/16.29
R2G-STNN	77.76/09.92	76.17/07.43	**82.30/10.21**	**88.35/10.52**	88.90/09.97	93.38/05.90	–
BiHDM	–	–	–	–	–	93.12/06.06	74.35/14.09
FGCN	**78.91/10.61**	**76.96/06.77**	77.64/12.44	87.13/06.39	**89.87/10.12**	**94.10/07.34**	**77.14/15.71**

**Table 2:** The accuracy and standard deviation on SEED with DASM features. The best results are marked in bold.

Classifier	SVM [31]	GCN [27]	DGCN [9]	Ours
δ (%)	48.87/10.49	57.07/06.75	55.93/09.14	**63.36/07.94**
θ (%)	53.02/12.76	54.80/09.09	56.12/07.86	**62.84/09.33**
α (%)	59.81/14.67	62.97/13.43	64.27/12.72	**66.72/12.08**
β (%)	75.03/15.72	74.97/13.40	73.61/14.35	**81.27/12.75**
γ (%)	73.59/16.57	73.28/13.67	73.50/16.60	**82.57/13.83**
Total (%)	72.81/16.57	76.00/13.32	78.45/11.84	**78.67/11.57**

**Table 3:** The accuracy and standard deviation on SEED with DACU features. The best results are marked in bold.

Classifier	SVM [31]	GCN [27]	DGCN [9]	Ours
δ (%)	55.92/14.62	62.60/12.88	63.18/13.48	**67.81/11.94**
θ (%)	57.16/10.77	**65.05/08.35**	62.55/07.96	64.47/08.98
α (%)	61.37/15.97	66.41/11.06	67.71/10.74	**67.73/12.81**
β (%)	75.17/15.58	77.28/11.55	78.68/10.81	**79.93/10.64**
γ (%)	76.44/15.41	18.68/13.00	80.05/13.03	**83.17/11.90**
Total (%)	77.38/11.98	79.02/11.27	81.91/10.06	**84.10/10.63**

**Table 4:** The influence of different fusion strategies on SEED dataset. The best results are marked in bold.

Fusion Strategy	Accuracy
Point-by-point Addition (%)	**94.10**
Point-by-point Product (%)	91.78
Kronecker Product (%)	90.56
Kronecker Addition (%)	91.41
Cross Diffusion Process (%)	90.36

## Data Availability

The datasets analyzed during the current study are available in the SEED repository, https://bcmi.sjtu.edu.cn/~seed/index.html (accessed on 2015).

## Abbreviations

The following abbreviations are used in this manuscript:

EEG	Electroencephalogram
DE	Differential entropy
DASM	Differential asymmetry
DCAU	Differential caudally
CNN	Convolutional neural network
